# Pancreatic cancer and exosomes: role in progression, diagnosis, monitoring, and treatment

**DOI:** 10.3389/fonc.2023.1149551

**Published:** 2023-05-23

**Authors:** Xingliang Fang, Huanrong Lan, Ketao Jin, Jun Qian

**Affiliations:** ^1^ Department of Hepatobiliary Surgery, Affiliated Hospital of Shaoxing University, Shaoxing, Zhejiang, China; ^2^ Department of Surgical Oncology, Hangzhou Cancer Hospital, Hangzhou, Zhejiang, China; ^3^ Department of Colorectal Surgery, Affiliated Jinhua Hospital, Zhejiang University School of Medicine, Jinhua, Zhejiang, China; ^4^ Department of Colorectal Surgery, Xinchang People’s Hospital, Affiliated Xinchang Hospital, Wenzhou Medical University, Xinchang, Zhejiang, China

**Keywords:** pancreatic cancer, exosome, diagnosis, prognosis, tumor, treatment, miRNA

## Abstract

Pancreatic cancer (PC) is one of the most dangerous diseases that threaten human life, and investigating the details affecting its progression or regression is particularly important. Exosomes are one of the derivatives produced from different cells, including tumor cells and other cells such as Tregs, M2 macrophages, and MDSCs, and can help tumor growth. These exosomes perform their actions by affecting the cells in the tumor microenvironment, such as pancreatic stellate cells (PSCs) that produce extracellular matrix (ECM) components and immune cells that are responsible for killing tumor cells. It has also been shown that pancreatic cancer cell (PCC)-derived exosomes at different stages carry molecules. Checking the presence of these molecules in the blood and other body fluids can help us in the early stage diagnosis and monitoring of PC. However, immune system cell-derived exosomes (IEXs) and mesenchymal stem cell (MSC)-derived exosomes can contribute to PC treatment. Immune cells produce exosomes as part of the mechanisms involved in the immune surveillance and tumor cell-killing phenomenon. Exosomes can be modified in such a way that their antitumor properties are enhanced. One of these methods is drug loading in exosomes, which can significantly increase the effectiveness of chemotherapy drugs. In general, exosomes form a complex intercellular communication network that plays a role in developing, progressing, diagnosing, monitoring, and treating pancreatic cancer.

## 1 Introduction

Pancreatic cancer (PC) is one of the most dangerous cancers related to the digestive tract, which ranks fourth and sixth in America and China, respectively, as the leading cause of cancer-related death (1). This disease symptoms usually do not appear until a large part of the pancreas is damaged, and the symptoms stage is too late to start treatment (2). The usual treatments for pancreatic cancer are surgery and chemotherapy (3). However, it seems that in most cases, after surgery, cancer recurs and can be dangerous to the patient’s life (4). In the meantime, tumor cells can suppress the related responses needed for tumor regression by affecting the immune system (5). Tumor cells usually perform this action through several mechanisms, which include the production of suppressive soluble cytokines, the expression of surface molecules, and the production of extracellular vesicles (EVs) (6, 7). EVs are expressed in two categories. The first category of classification is based on cell origin, how to separate from the cell, size, and contents; they are divided into three subclasses, namely, apoptotic bodies, microvesicles, and exosomes. In the new classification, EVs with a maximum diameter of 200 nm are classified as small EVs (sEVs) and EVs larger than 200 nm as medium/large EVs (m/lEVs). However, in articles that investigate the therapeutic properties of vesicles, authors usually use the word exosomes as one of the subfamilies of sEV. Due to the characteristics of exosomes, such as biocompatibility, low immunogenicity, small size, ability to pass through small vessels, and ease of isolation and storage, they have received much attention in the field of tumors.

Exosomes are double-layered, nano-sized vesicles that are produced by almost all cells, and their role is to maintain homeostasis and intercellular communication (8). These vesicles carry various components (proteins, lipids, and nucleic acids), and after reaching the target cells, they transfer these cargos to them by different methods, including fusion, and change the characteristics of the target cells (9). Like other cells, tumor cells communicate with the cells in the tumor microenvironment through the production of exosomes and change their features in a useful way for tumor growth (10). However, exosomes produced from tumor cells at different stages have different components (including different microRNAs) that can be used as potential markers for diagnosing and monitoring the pancreatic cancer treatment process, progress, and regression (11, 12). Therefore, the importance of investigating and isolating exosomes derived from tumor cells is increasing daily because they are suitable biological tools for understanding the tumor stage.

**Figure 1:**
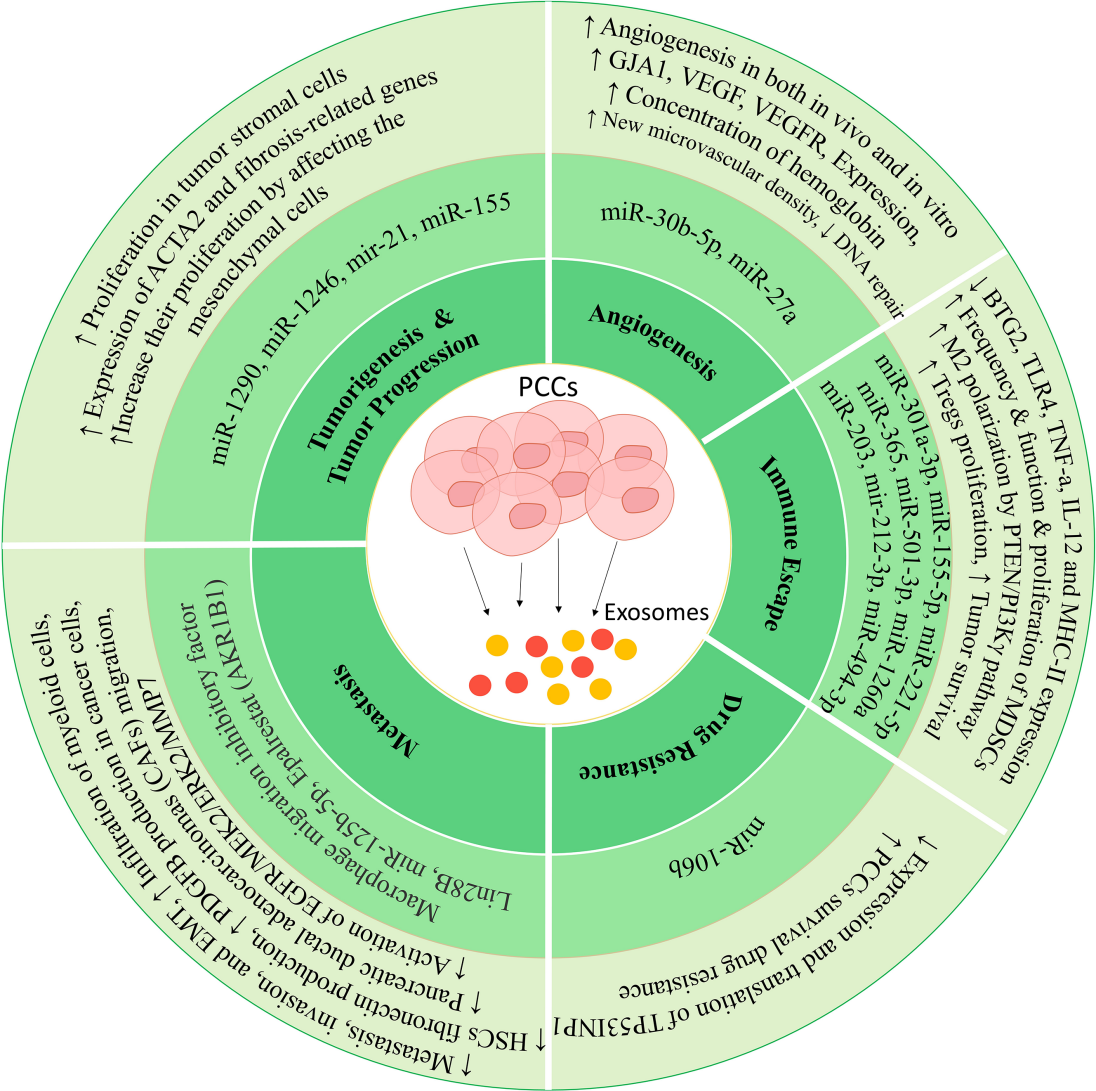
Mechanisms in which exosomes can lead to tumor progression. These vesicles can increase proliferation, invasion, metastasis, drug resistance, and immune deviation by affecting different cells.

Meanwhile, exosomes derived from other cells, including immune cells, can be used as therapeutic agents in all tumors (13, 14). Moreover, investigation of tumor behavior in the therapeutic use of mesenchymal stem cell (MSC)-derived exosomes is very important due to the presence of these cells in most tumor tissues and their effect on the treatment outcome (15). Today, different methods are used in which the therapeutic potential of exosomes has increased, such as drug- and various miR-loaded exosomes and engineered exosomes for targeted delivery (16), which lead to better tumor regression compared with intact exosome applications (17). Therefore, examining studies that reveal the relationship between exosomes and PC is of particular importance and can lead to a better orientation of researchers toward new treatments based on exosomes and the early diagnosis of this cancer. In the following, we will discuss important aspects of the relationship between pancreatic cancer and exosomes, including the role of exosomes in progression and metastasis, their diagnostic applications, and their therapeutic uses (**Figure 1**).

## 2 Tumor-derived exosomes in pancreatic cancer progression

Tumor cells, like other cells, produce EVs for intercellular communication and cell homeostasis maintenance. After being produced from a tumor cell, these vesicles can have an autocrine effect on the cell itself or a paracrine and endocrine effect on the cells in the tumor microenvironment or other tissues and lead to the formation of a tumor nano environment (TNE) (18). EVs are divided into different subtypes based on their size and formation model (19). Exosomes are more prevalent in laboratory and clinical applications due to their advantages, such as small size, biocompatibility, and manipulation capabilities (20). Today, it is said that exosomes should be called sEV because the exosome population produced from a cell or a set of cells is heterogeneous and does not all reflect a specific characteristic (21). Usually, the characterization of sEVs is complex, and according to MISEV2018, the EV source and preparation should be described quantitatively (22). Different characteristics of sEVs should be investigated to confirm their separation. For example, the size of the vesicles should be checked using dynamic light scanning (DLS) and their shape by electron microscopy (SEM and TEM). Also, these vesicles should be evaluated to examine the expression of surface markers such as CD83, CD63, CD9, and integrin, as well as intracytosolic markers such as ALIX, TSG101, syntenin, and HSP70 (22). The components of sEVs can be different based on the origin and biological functions of the producing cell. Various components of exosomes can change under cell conditions. For example, the upregulation of oncogenes in cancer cells can increase their exosome oncoprotein levels, such as HRS and EGFR type III (23). sEVs are captured by the target cell through several biological mechanisms, including fusion, pinocytosis, phagocytosis (macrophage and myeloid cells), receptor-mediated endocytosis, caveolae-mediated endocytosis, and lipid raft-mediated endocytosis (24). One of the main routes by which tumor-derived sEVs are removed by the target cell is receptor-mediated endocytosis, because these vesicles have ligands such as FASL, PD-L1, and TRAIL on their surface, which increases endocytosis (25). Also, the expression of growth factors and their binding to EGFR can stimulate the harvesting of sEVs through pinocytosis (26). In congruence with the latest suggestion, we refer to “exosomes” as “sEVs” (22) throughout this review.

Exosomes produced from tumor cells by affecting the target cells can facilitate the conditions for tumor cell proliferation, metastasis, drug resistance, tumor-specific immune suppression, and angiogenesis, and can actually lead to tumor progression (27). Exosomes usually affect their target cells in three ways. 1) After the release of exosomes and their movement toward the target cells, their surface molecules bind to the surface receptors of the target cell and lead to signal transduction through these receptors (juxtacrine signaling). 2) In some cases, the exosome surface molecules are cut adjacent to the target cell and then bind to their receptor and exert their effects (soluble signaling). 3) In the third method, which is more common than the previous two methods, exosomes are integrated with the target cell using various mechanisms such as fusion and receptor-mediated endocytosis or phagocytosis, and transfer their internal content (protein, lipid, nucleic acid) to it, which also leads to changing the responses and conditions of the target cell (28, 29). In new studies, it is said that exosomes, in addition to the usual cargo, can also transfer mitochondria to the target cell (30). Since the mitochondria play an important role in metabolism and maintaining cell proliferation, transferring mitochondria via exosomes to tumor cells can increase their proliferation ability (31, 32). It has also been shown that the transfer of mitochondria from MSCs by exosomes can increase the chemoresistance ability of tumor cells (33). Depending on the origin of the exosome, different responses can be initiated in the target cell. For example, exosomes derived from immune system cells can lead to the expansion or suppression of tumor growth based on their origin (34, 35). Due to their immunomodulatory properties, the presence of MSC-derived exosomes can lead to tumor progression (36). Also, exosomes produced from tumor cells and released into the tumor environment can suppress the responses of the immune cells and, by affecting the endothelial cells, increase the angiogenesis and mobility, migration, and metastasis of tumor cells (37, 38).

### 2.1 Tumorigenesis and tumor progression

Exosomes produced from pancreatic cancer cells (PCCs) differ from exosomes produced from normal pancreatic cells in terms of content and heterogeneity (39). Exosomes derived from tumor cells can transfer the oncopeptide of oncoproteins to the target cells, leading to phenotypic changes and tumorigenesis of the target cell (40). It has also been shown that people with tumors have more exosomes in their blood than healthy people, indicating that more exosomes are produced from tumor cells than from healthy cells (41). The results of the studies show that these exosomes can act as an initiator for the random mutations that occur in the target cell and lead to the transformation of a healthy cell into a tumor cell. Of course, it is worth mentioning that transforming a cell into a cancerous state requires an initiator and a promoter.

In a study conducted by Stefanius and colleagues, it was shown that using exosomes derived from PCC lines can lead to mutations in the KRAS gene (a proto-oncogene) and to the transformation of NIH/3T3 cells that have a mesenchymal origin. This study was conducted in two phases, *in vivo* and *in vitro*, and showed that if tumor cell exosomes were used as initiators and cadmium chloride (CdCl) as a promoter, the rate of tumor formation and cell transformation increased significantly compared with the control (42). It also leads to fundamental changes in the proteomics of these cells. Therefore, it was found that transferring various cargoes from tumor cells to healthy cells can lead to tumorigenesis. Among the different cargoes, non-coding RNAs play an important role in exosome applications. For example, exosomes derived from PCCs stimulate pancreatic stellate cells (PSCs) (43). These stimulated PSCs produce exosomes that contain miRNA-21, which can be transferred to other cells in tumor tissue, such as the pancreas, and alter their actions, leading to increased proliferation in tumor stromal cells (44). Cancer cells seem to activate PSCs by producing exosomes containing miRNA-1290 and miRNA-1246 and releasing them into the tumor environment (45). Also, exosomes derived from tumor cells have miRNA-155 by themselves, which can increase their proliferation by affecting the mesenchymal cells in the tumor environment (46). These mesenchymal cells and mesenchymal stem cells derived from bone marrow can differentiate into fibroblastic cells via cancer-derived exosomes, which play an important role in supporting the proliferation of pancreatic cancer cells (47).

### 2.2 Metastasis

Metastasis is a complex process that occurs via a set of cells and different factors in the tumor environment and is usually accompanied by neoangiogenesis (48, 49). This angiogenesis leads to the migration of tumor cells to distant places and their implantation and development in a new location (50). In a study, it has been shown that the expression level of Aldo-keto reductase family 1 member B1 (AKR1B1) in pancreatic cancer cells is associated with an increased level and release of exosomes from tumor cells (51). Moreover, previous studies show that epalrestat, which is an inhibitor of AKR1B1, can be used as an inhibitor of metastasis in different types of tumors (52). Considering that AKR1B1 leads to negative regulation of lysosome trafficking to endosomes and increases the formation of multivesicular bodies (MVBs) in cancer cells (53), the application of drugs such as epalrestat leads to a decrease in metastasis and reduces the amount of exosome production from cancer cells. Therefore, it can be concluded that the production of exosomes by PCCs can be a potential pathway to stimulate metastasis-related mechanisms.

Proteomic analysis of PCCs and normal pancreatic cell- derived exosomes shows that PCC exosomes carry various key factors regulating metastasis and molecules related to signaling required for metastasis and can lead to increased metastasis. Identifying the pathways leading to metastasis is crucial because cancer progression can be prevented by inhibiting these mechanisms. In the case of pancreatic cancer, one of the cells that play a role in the progression of metastasis is pancreatic ductal adenocarcinomas (PDACs) (54). These cells help tumor metastasis by producing exosomes that contain a large amount of macrophage migration inhibitory factor (MIF) (55, 56). These exosomes migrate to the liver, and after uptake by Kupffer cells, lead to the production of TGF-β from them (57). This cytokine affects hepatic stellate cells (HSCs) and leads to their activation, which is associated with the production of various components of the extracellular matrix and fibrosis of the liver tissue (58). The important point is that inhibiting MIF transfer by exosomes or using anti-TGF-β inhibits pancreatic cancer metastasis to the liver (57, 59). All these results show that exosomes derived from PDACs can play a role as a potential pathway in the metastasis of this cancer. One of the important and decisive components of exosomes as metastasis-regulating agents is the expression of various integrins through these vesicles. Integrin alpha V beta 5 (ITGavb5)-expressing exosomes bind specifically to Kupffer cells and induce tropism of these vesicles to the liver tissue. Other integrins, including ITGa6b4 and ITGa6b1 on sEVs, can bind to epithelial cells and resident fibroblasts in the lung and increase their tropism to the lung tissue (60–62).

Another study showed that exosomes derived from PCCs induce the ability to form premetastatic niches and tumor growth in their livers after being injected into normal mice (63). Flow cytometric studies show that these exosomes can lead to increased expression and activity of the transcription factor STAT3, as well as increased infiltration of myeloid cells, leading to the activity of HSCs in the liver and increased production of fibronectin from them (63). The injection of PCC-derived exosomes leads to an increase in the frequency of myeloid-derived suppressor cells (MDSCs) in the peripheral blood of mice (63). As immune response suppressors, these cells can help tumor growth and spread (64). It was also found that these exosomes can lead to a decrease in the adhesion of Panc02 cells and an increase in their migration, which increases their metastatic and invasive potential (63). Therefore, in addition to PDACs, pancreatic tumor cells can help tumor spread by increasing their metastatic properties by producing exosomes.

In addition to the mentioned cases, some studies have confirmed the presence of PSCs in metastatic tumors derived from pancreatic cancer. One of the studies investigated metastatic cells’ ability to induce the migration of pancreatic ductal adenocarcinomas (CAFs) and PSCs (as the main factor in tumor expansion) (65). The results of this study show that exosomes produced by PCCs stimulate the migration of these cells. Specific pathways have been proposed for this phenomenon. Still, one of the most important pathways is mediated by the exosomal protein Lin28B, and it was proven by Yue-Feng Zhang et al. that fusion of the PCC-derived exosomes by PSCs leads to the activation of the Lin28B/let-7/HMGA2/PDGFB-related pathway, which is normally associated with increased production of PDGFB in cancer cells and inhibits itself through miR let-7 expression (65, 66). Since the expression of miR let-7 is inversely correlated with the expression of HMGA2/PDGFB (67), the suppression of miR let-7 expression leads to increases in the expression of the HMGA2/PDGFB (65). Since PDGFB is a well-known chemokine, it seems that this molecule can help with PSCs and their migration to the secondary site of tumor formation during metastasis (68, 69). The important point is that Western blot analysis indicates the absence of PDGFB inside exosomes, and an increase in its expression was shown after the fusion of PCC-derived exosomes by PSCs (65).

As mentioned before, exosomes produced by cells have different contents in different conditions (70). miRNA sequencing of exosomes derived from PC-1.0 cells (prometastatic cells associated with pancreatic cancer) (71) and PC-1 (as weakly invasive cells) showed that these exosomes differ in the amount of 62 miRNAs, and miR-125b-5p is highly upregulated in the exosomes of cells with high metastasis ability (72). Functional investigations show that these miRNAs play an important role in increased metastasis, invasion, and epithelial-to-mesenchymal transition (EMT) (73). Moreover, investigating the miR-125b-5p mechanism of action showed that this miRNA binds to STARD13, which plays an essential role in the good prognosis of pancreatic cancer (72). Adding PC-1.0-derived exosomes to weakly invasive pancreatic cancer cells increases their ability in metastatic processes. Therefore, these exosomes seem to increase the metastatic capacity of PC-1 cells by carrying miR-125b-5p and its binding to STARD13. The subsequent suppression of STARD13 expression in weakly metastatic PC-1 cells leads to the activation of EGFR/MEK2/ERK2/MMP7 signaling pathways related to metastasis in different cancer cells (72).

### 2.3 Angiogenesis

Since tumor cells grow more than the other cells in the tumor site, most of the oxygen is consumed by these cells (74). Usually, a hypoxia condition is established in the tumor microenvironment, which can ultimately lead to neoangiogenesis (75). A study published by Kai Chen et al. in 2022 showed that the amount of miR-30b-5p in exosomes derived from hypoxic PCCs is increased (76). After being transferred to endothelial cells by exosomes, this exosomal miRNA binds to the 3′UTR region and inhibits the expression of a gap junction-related protein called GJA1, which can lead to tube formation, increasing the migration of endothelial cells and ultimately increasing angiogenesis (76). The results of the *in vivo* phase in animal studies also confirm the results of the *in vitro* phase, in which the group receiving the exosomes derived from hypoxic PCCs had a higher hemoglobin concentration than the group treated with PBS. Moreover, the results of immunohistochemistry analysis showed that the injection of these exosomes increased the new microvascular density by increasing the population of CD31^+^ cells (76). In another study, microarray analysis showed an increase in another miRNA called miR-27a (77). In this study, exosomes derived from the PANC-1 cell line (pancreatic cancer cell line) were used to treat human microvascular endothelial cells (HMVECs), and their effects on invasion, proliferation, and angiogenesis were investigated. Moreover, the *in vivo* phase was performed with xenograft injection in nude mice. The results of this study show that exosomal miR-27a leads to the negative regulation of B-cell translocation gene 2 (BTG2) and is associated with increased angiogenesis in both *in vivo* and *in vitro* conditions (77). BTG2 is a molecule involved in DNA repair and has an antitumor role (78). Western blot analysis after the co-culture of exosomes derived from PANC-1 with HMVEC shows an increase in the production of proteins, such as VEGF, VEGFR, MMP-2, and MMP-9 in HMVEC, related to angiogenesis and metastasis (77).

### 2.4 Tumor immune escape

After the formation of tumor cells, the immune system can eliminate them by identifying new antigens (neoantigens) produced by tumor cells (immune surveillance) and preventing their spread (79). However, immune responses are usually suppressed in these areas due to the specific conditions of the tumor microenvironment (80). Most immune cells in this area are Tregs (81), M2 macrophages (82), and MDSCs (83). The inhibiting of the immune system occurs by various mechanisms, including direct contact between cells and inhibition by surface molecules such as CTLA-4 and PD-1 (84), as well as the production of cytokines that inhibit immune system responses, such as IL-10 and TGF-β (85). However, it has been shown that the transfer of exosomes from tumor cells to immune cells located at the tumor site can inhibit their responses (86). Macrophages around the tumor are known as tumor-associated macrophages (TAMs) and are derived from monocytes migrating to the tumor site (87), which have immune-suppressive effects (88). In this place, macrophages capture PCC-derived exosomes, leading to their polarization to the M2 phototype, which produces pro-tumorigenic factors (89). A study showed that exosomes derived from hypoxic PCCs by transferring miR-301a-3p to macrophages stimulate their polarization to M2 phenotype through the PTEN/PI3Kγ pathway (90). It has also been shown that exosomes derived from pancreatic tumor cells enriched with ezrin after inducing M2 polarization can increase tumor metastasis to the liver (91). On the other hand, when M2 macrophages are found in the tumor site, they can help tumor expansion by producing exosomes (92). Exosomes derived from M2 macrophages induced in the pancreatic tumor site containing various cargoes such as miR-155-5p, miR-221-5p (93), miR-365 (94), and miR-501-3p (95) contribute to tumor survival by influencing different pathways. For example, M2 exosomes containing miR-365 can suppress BTG2 expression. Since BTG2 plays a role in inhibiting the FAK/AKT pathway, therefore, inhibiting its expression leads to the activation of the FAK/AKT signaling pathway and increases the development of pancreatic cancer (94). Thus, PCC-derived exosomes lead to the suppression of immune responses in macrophages, and in this way, they can also increase their survival and progression.

In addition to macrophages, MDSCs also play a role in tumor-induced immune escape (96). Continuous stimulation signals and hypoxia at the tumor site lead to the differentiation of these cells into TAM-like cells (97), which have high pro-tumorigenic activity. Various studies have shown that PCC-derived exosomes containing miR-1260a and miR-494-3p can affect different populations of MDSCs and lead to increased proliferation and their immunosuppressive ability (98). In addition to the effect of PCC exosomes on the innate immune system cells, these exosomes can also affect the responses of T cells in two ways, direct and indirect (impact on antigen-presenting cells) (99). A study has shown that dendritic cells (DCs), after uptake of PCC-derived exosomes through the transfer of miR-203, lead to a decrease in the expression of TLR4, TNF-a, and IL-12, and by transferring mir-212-3p to this cell the MHC-II expression in DCs is reduced (100, 101). Therefore, exosomes derived from PCCs can reduce the ability of DCs to induce and initiate strong T-cell responses (102). In one of the new studies, it has been shown that exosomes derived from PCCs can increase the proliferation ability of Tregs (103). This study showed that SIRT1, SIRT2, ATM, AMPK, and SIRT6 were sequentially activated in T lymphocytes treated with exosomes (103). As the most important suppressive immune cells, these cells help inhibit immune responses by producing various cytokines, absorbing IL-2 in the environment, cell-to-cell contact, etc. (104). In general, it can be said that PCCs communicate with immune cells by exosomes in a two-way manner and can lead to tumor expansion and failure of immune surveillance.

### 2.5 Tumor drug resistance

One of our main problems in chemotherapy is the drug resistance of cancer cells (105). Gemcitabine (GEM) is one of the primary drugs involved in the chemotherapy of pancreatic cancer (106), whose application for a long time has led to the acquisition and expansion of drug resistance in tumor cells (107). Some other cells, including CAFs, can play a role in developing this drug resistance (108). A study by Yuan Fang et al. investigated the role of these fibroblasts and their exosomes in the drug resistance of PCC (109). The results of this study show that fibroblasts producing exosomes containing miR-106b can induce this phenomenon in PCCs (109). Moreover, the pretreatment of fibroblasts with miR-106b production-inhibiting factors leads to a decrease in the amount of miR-106b in exosomes and a decrease in the drug resistance of PCCs to gemcitabine (109). To understand the potential mechanisms where miR-106b leads to an increase in the drug resistance of PCCs, different targets of this miR were investigated, and it was found that this microRNA plays an essential role in the drug resistance of breast cancer by targeting the tumor protein p53-inducible nuclear protein gene 1 (TP53INP1) by binding to the messenger RNA (mRNA) 3′ end of this gene. The expression of this gene is regulated by factors such as p53, p73, and E2F1, and its product has two isoforms that play an important role in the function of p53 (110). Moreover, to confirm this, it was shown that overexpression of TP53INP1 leads to the reversal of the miR-106b function (109). Therefore, it can be said that exosomes derived from CAFs, by transferring miR-106b to PCCs, lead to the inhibition of the expression and translation of TP53INP1-related mRNA, which leads to an increase in their survival and gemcitabine drug resistance in PCCs.

Another study showed that the amount of five microRNAs was higher than others in CAF-derived exosomes, namely, miR-222, miR-221, miR-181a, miR-92a, and miR-21 (111). After being transferred to PCCs, investigations show that these microRNAs can increase chemoresistance to gemcitabine in the PCCs by inhibiting the expression and production of phosphatase and tensin homolog (PTEN)-related protein *in vitro* (111). PTEN plays an essential role in suppressing tumor formation (112). The reduction of its expression and the mutation in its gene are observed in many tumors, leading to uncontrolled tumor proliferation and expansion (113). Therefore, by producing these exosomes, CAFs contribute to the drug resistance of tumor cells and lead to cancer progression. To further confirm the role of CAF-derived exosomes in this study, it was shown that GW4869 treatment *in vivo* led to the inhibition of exosome production and decreased drug resistance (111). The tumor size in GW4869-treated mice was not associated with a significant increase compared to control mice, which indicates the importance of exosomes in the drug resistance of tumor cells and their cross-talk with other cells (111).

## 3 Exosomes in pancreatic cancer diagnosis and monitoring

As mentioned earlier, exosomes (especially PCC-derived exosomes) are produced more in pancreatic cancer patients, and exosome serum levels are significantly increased compared with healthy individuals and non-pancreatic cancer patients (114). Therefore, according to the presence of these exosomes in the serum, their increase in pancreatic cancer, and their detection capability, they can be used in diagnosing and monitoring agents in PC (115). Apart from CA-199, which has FDA approval for the diagnosis of pancreatic cancer (116), PC lacks molecular biomarkers for early diagnosis, and the definition of a diagnosis system can significantly help reduce PC mortality worldwide (117). In the case of exosomes, RNA (especially micro RNAs) is usually used for diagnosis (115). Compared to free RNAs in peripheral blood, they have more diagnostic value in terms of quantity and quality (118–120). In addition to serum, plasma, and peripheral blood, other body fluids, such as pancreatic juice and saliva, can be used for exosome isolation and pancreatic cancer diagnosis (121). In addition, in some cases, exosomes derived from other cells (such as NK cells and macrophages) (95, 122) can help in the diagnosis of pancreatic cancer because they are involved in the responses related to the expansion or non-expansion of cancer. Usually, different exosomal miRNAs can help in tumor stage diagnosis, early diagnosis, prognosis assessment, drug resistance, tumor cell viability, and tumor recurrence after treatment. **Table 1** summarizes the details of some exosomal miRNAs detected in recent studies (**Table 1**) The important point about the diagnostic use of exosomal miRNAs is that they play a significant role in the maintenance, expansion, proliferation, and invasion of pancreatic cancer, as mentioned previously (130). In addition to miRNAs, other components of exosome internal contents such as proteins, mRNAs, long non-coding RNAs (lncRNAs), and circular RNAs (circRNAs) can also be used as diagnostic tools (**Table 2**) (143).

**Table 1:** Exosomal miRNA role in the diagnosis of prostatic cancer (PC).

Exosomal cargo	Cargo type	Origin or cell source	Biomarker or other roles	Number of patients	Year	Ref.
Glypican-1	Protein (heparan sulfate proteoglycans)	Serum	1) Diagnostic biomarker for dividing PC 2) Benign pancreatic disease biomarker 3) Prediction of prognosis	190	2015	(123)
Exosomal integrin	Protein (adhesion)	N/A	Prognostic	27	2010	(124)
Migration inhibitory factor (MIF)	Protein (cytokine)	1) Plasma 2) Serum	Liver metastasis prognostic marker	40	2015	(61)
Sox2ot	lncRNA	Peripheral blood	Early diagnosis	61	2018	(125)
MALAT-1	lncRNA	Peripheral blood	Early diagnosis	N/A	2020	(126)
HULC	lncRNA	Peripheral blood	Early diagnosis	N/A	2020	(126)
GPC1	mRNA	Peripheral blood	1) Early PC diagnosis 2) Tumor stage	133	2017	(127)
WASF2	mRNA	Serum	1) Early PC diagnosis 2) Tumor stage	27	2019	(128)
IARS	circRNA	1) Culture medium 2) Peripheral blood	1) Tumor stage 2) Survival evaluation	92	2018	(129)

**Table 2:** Role of other types of exosomal cargos in pancreatic cancer diagnosis.

Exosomal miRNA	Origin or cell source	Biomarker or other roles	Number of patients	Year	Ref.
miR-17-5p	Serum	1) Diagnostic for PC advanced stage 2) Metastasis	49	2013	(131)
	Plasma	Diagnostic biomarker for dividing PC and non-PC	20	2015	(132)
miRNA-21	1) Portal vein blood (serum) 2) Pancreatic juice	1) Diagnostic chronic pancreatitis 2) Early diagnostic 3) Prognostic 4) Recurrence diagnostic 5) Tumor survival evaluation	49	2013	(131)
			55	2019	(133)
			35	2019	(121)
miRNA-210	1) Peripheral blood	1) Early diagnosis 2) Tumor stage	40	2020	(134)
	2) PSC	Prognostic (gemcitabine resistance)	*In vitro*	2020	(135)
miRNA-10b	Peripheral blood (serum)	Early diagnosis	36	2020	(136)
			40	2017	(137)
miRNA-3976	Serum	Early diagnosis	190	2015	(138)
miR-222	1) Peripheral blood 2) PCC	1) Tumor stage diagnosis 2) Survival evaluation 3) Prognostic	Cell line/*in vitro*	2018	(139)
miRNA-1246	Peripheral blood (serum)	Early diagnosis	15	2017	(139)
miRNA-451a	1) Portal vein blood	1) Recurrence diagnostic 2) Tumor survival evaluation	55	2019	(133)
	2) Plasma	Prognostic and recurrence	46	2018	(140)
miRNA-155	1) Pancreatic juice	1) Early diagnosis 2) Prognostic	35	2019	(121)
	2) PCC	Prognostic for gemcitabine resistance	1) *In vitro*	2017	(141)
			2) *In vivo* = 45		
miRNA-4644	Saliva	Early diagnosis	12	2016	(142)
miR-3607-3p	NK cells	Prognostic	40	2019	(122)

## 4 Therapeutic applications of exosomes in pancreatic cancer

**Figure 2:**
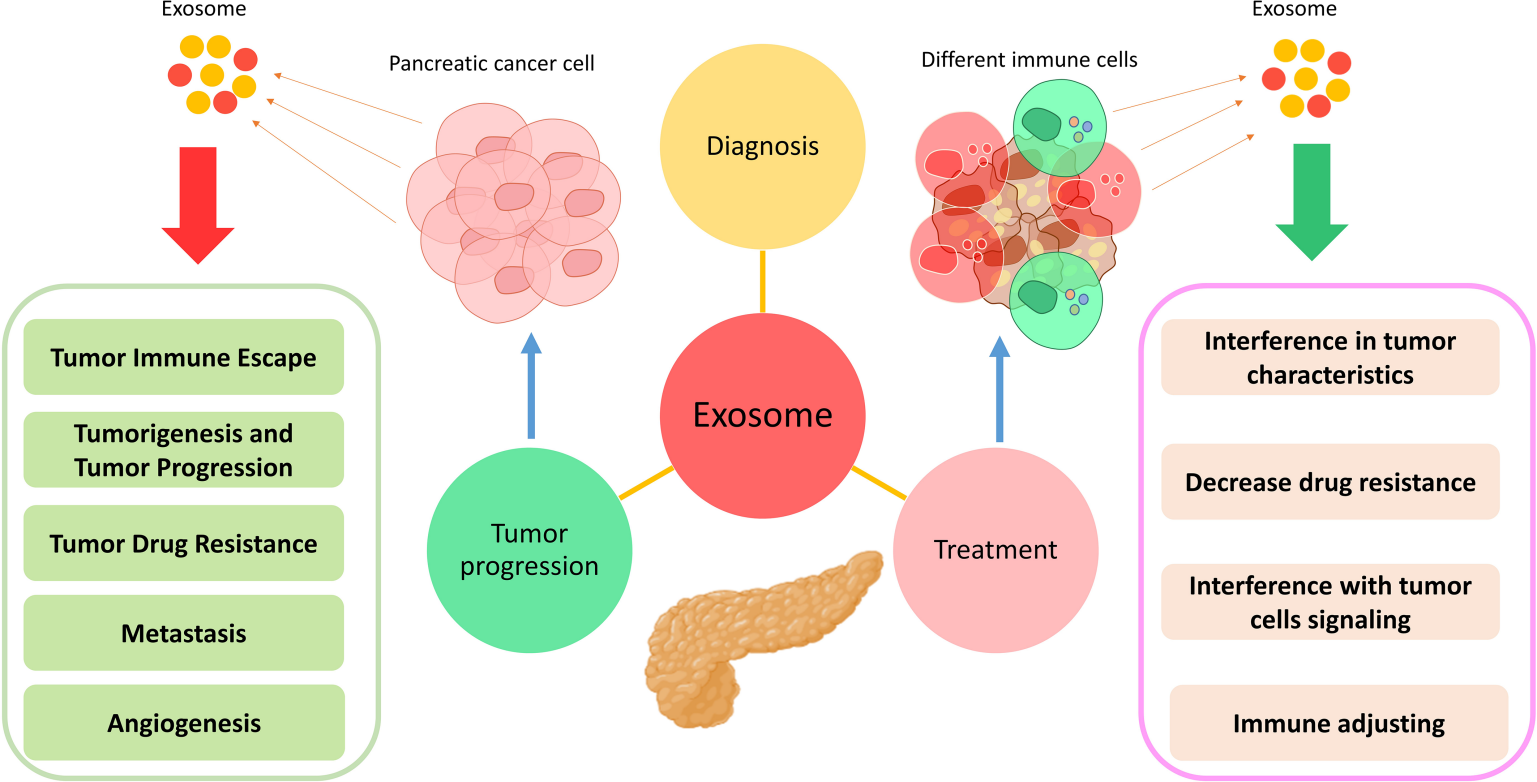
Application of exosomes in the diagnosis, treatment, and progression of pancreatic cancer. Exosomes exert their effects by changing various characteristics in different types of cells.

Pancreatic cancer is usually treated by chemotherapy, radiotherapy, and surgery (144). However, each treatment has different side effects that can affect and even threaten the patient’s life (145). Therefore, it is essential to use methods that reduce the dose and application times of these treatments. *In vitro* results show that using exosomes can reduce the effective dose of antibiotics used in infectious diseases (146). Moreover, the effectiveness of these vesicles in treating other conditions, such as autoimmune diseases, transplant rejection, neurodegenerative diseases, infectious diseases, and liver-related diseases, has been proven (147–149). In the meantime, the use of exosomes has been widely used in the treatment of various cancers (92). The results of studies have shown that successive injections of exosomes do not have any toxic effect on the body, their size is so small that they can pass through various vascular and capillary barriers, and they have immunocompatible and biocompatible properties suitable for use in therapeutic applications (20, 150). For these reasons, the application of exosomes has more priority than cell therapy. The antitumor role of exosomes derived from immune system inflammatory cells, including DCs, macrophages, NK cells, and T cells, has been well demonstrated (92). However, exosomes in the treatment of cancers are used in two ways: in the first case, exosomes in their naive and intact form are used (151), and in the second case, exosomes act as drug carriers (152). Moreover, the use of engineered exosomes, known as iExosomes, has increased in recent years. In fact, exosomes that contain loaded drugs, microRNAs, siRNAs, and CRISPR/Cas system components are considered part of this therapeutic approach (153). In some cases, exosomes are manipulated by surface molecules in order to increase therapeutic efficiency. For example, iExosomes can be engineered to express a chemokine receptor to a greater extent and migrate to the desired site in a targeted manner (154). Exosomes can perform their therapeutic function by affecting various mechanisms (**Figure 2**).

### 4.1 Adjusting immunity

DC-derived exosomes (DEXs), which have been previously primed with pancreatic cancer-derived antigens, contain specific MHC-I/II–peptide complexes that can trigger antitumor responses in CD4^+^ and CD8^+^ T cells (155, 156). In addition, these exosomes can lead to the maturation of immature DCs, which is associated with the production of various cytokines and the activation of tumor-specific T cells (157, 158). Moreover, other studies have shown that heat shock proteins (HSPs) carried by exosomes can reduce the activation threshold and lead to the activation of T cells (159). Another study used DCs loaded with PCC-derived exosomes for pancreatic cancer treatment (159). Considering that exosomes derived from PCCs are a suitable source for various tumor antigens, DCs, after harvesting these exosomes, provide peptides and other tumor antigens and can help T cells to perform antitumor activities. Moreover, the results of this study show that intratumoral injection of these DCs can lead to modulating the responses of MDSCs and Treg cells (159).

### 4.2 Interference with tumor cell signaling

Sixty-three different mutations have been identified in somatic genes (160); 50% of them are associated with tumor-suppressor genes such as *KRAS*, *TP53*, *SMAD4*, and *CDKN2A* (161). *KRAS* is a mutation in 90% of pancreatic cancer patients during which a substitution in the 12th amino acid (replacement of glycine with aspartate) has occurred (*KRAS^G12D^*), and this mutation plays an essential role in tumor proliferation and metastasis (162). In a study by Kamerkar et al., human foreskin fibroblast cells were used for exosome isolation and therapeutic application (163). Investigations show that these exosomes have shRNA or siRNA that inhibits the expression of oncogenic *KRAS*. The results of this study show that the expression level of *KRAS^G12D^* in the pancreatic cancer cell line treated with these exosomes has decreased (163). Moreover, animal studies have demonstrated the reduction of metastasis in these models (163). In addition, exosomes can be used as carriers (164). Due to their characteristics, MSC-derived exosomes are usually suitable drug carriers for various therapeutic purposes (15). In a study by Yujin Lee et al., after exosomes were isolated from the supernatant of umbilical cord-derived MSCs, miR-145-5p was loaded into them, and these exosomes were intratumorally administered to mice (165). This microRNA is associated with decreased pancreatic cancer tumor cells (166, 167) and exerts its antitumor effects by affecting TGF-β/Smad3 pathways (168). The results of this study also show that these exosomes are attached to the Smad3 3′-UTR after being captured by tumor cells by endocytosis [investigated by Luciferase (Gluc) activity assays] and lead to the reduction of xenograft tumor growth according to the immunohistochemistry and TUNEL assay results in both *in vivo* (BALB/c nude mice) and *in vitro* conditions (165). Moreover, in another study, exosomes have been used as CRISPR/Cas9 system DNA plasmid carriers to target *KRAS^G12D^* (169, 170). Exosomes loaded with CRISPR/Cas9 can target the mutated *Kras^G12D^* oncogenic allele in PCCs, thereby leading to the reduced proliferative ability of cancer cells and suppression of tumor growth in simultaneous subcutaneous and orthotopic models (169, 171). Therefore, exosomes can be used as promising carriers in pancreatic cancer gene therapy (169). Another gene whose overexpression is associated with pancreatic cancer is P21-activated kinase 4 (*PAK4*), which is associated with increased proliferation, survival, migration, and metastasis of these tumor cells (172, 173). Therefore, in a study, PCC-derived exosomes were isolated by ultracentrifugation, and siRNA was loaded into them by electroporation (172). In the next step, the colocalization of exosomes into PANC-1 cells was confirmed by fluorescent microscopy. The results of this study show that using these exosomes *in vivo* (intratumor injection) and *in vitro* leads to a decrease in PAK4 expression, a reduction in tumor growth, and an increase in the survival of mouse models (172). Moreover, the results of H&E staining show extensive apoptosis of tumor cells (172).

### 4.3 Decreasing drug resistance

Exosomes can be used as carriers for delivering chemotherapy drugs with high efficiency and biocompatibility characteristics (**Table 3**) (179). Since gemcitabine is used as the first line of chemotherapy in pancreatic cancer (180), in a study conducted in 2020 by Yong-Jiang et al., this drug was loaded into autologous exosomes derived from PANC-1 cells (ExoGEM), and its therapeutic efficiency was evaluated *in vivo* and *in vitro* (181). The animal model was created through intraperitoneal injection of PANC-1 cells into BALB/c nude mice, and exosomes were injected intravenously (182). The results of this study show that drug loading in exosomes has led to the improvement of gemcitabine’s release profile, delivery, cell absorption, and therapeutic efficacy (181). Therefore, due to the increase in drug efficiency in combination with exosomes, the use of these exosomes led to a significant decrease in tumor growth and increased survival of mice in a dose-dependent manner (181). The interesting point is that the use of these exosomes led to the reduction of side effects associated with chemotherapy drugs and led to targeted tumor therapy in mice.

**Table 3:** Encapsulated exosomes in efficient chemo drug delivery.

Drug	Exosome source	Cell line/animal model	Loading method	Result	Year	Ref.
Gemcitabine monophosphate (GEMP) and paclitaxel	BM-MSCs	1) MiaPaca-2 cells 2) 3D tumor spheroids 3) Nude mice	Electroporation	1) ↑ Endocellular dose of GEMP 2) ↑ Uptake of GEM, especially GEMP 3) ↑ Therapeutic efficacy 4) ↓ Cancer proliferative activity and chemoresistance	2020	(174)
Oxaliplatin	BM-MSCs	1) PANC-02 cells 2) C57BL/6 mice	Electroporation	1) ↑ Cellular uptake efficiency 2) ↑ Cytotoxicity and immunogenic cell death response 3) ↑ *In vivo* antitumor efficacy and enhanced immunity	2021	(175)
Gemcitabine	BM-MSCs	CFPAC-1 cells	Priming of MSCs	1) ↓ Cancer-related cell proliferation by 70% to 80% 2) Inhibit HUVEC proliferation	2015	(176)
Gemcitabine and deferasirox	M1 macrophage	1) PANC-1 cells 2) 3D tumor spheroids	Electroporation	1) ↑ Iron removal efficacy 2) Reverse drug resistance 3) ↑ hENT1 expression for the transport of drug combinations into cells 4) ↑ Accumulation of chemotherapeutic agents in the cytosol 5 ) ↓ Invasiveness of GEM resistance	2021	(177)
Curcumin	1) PANC-1 2) MIA PaCa-2	1) PANC-1 2 ) MIA PaCa-2	Methanol sonication	↓ Pancreatic adenocarcinoma cell viability	2015	(178)

### 4.4 Interference in tumor characteristics

The results of the studies show that the amount of miR-1231 in exosomes isolated from the peripheral blood of PC patients is associated with a significant decrease compared with healthy individuals (183). Moreover, the presence of miR-1231 in exosomes derived from bone marrow mesenchymal stem cells has been proven (184). It is also possible to increase the expression of this miRNA and its transport into exosomes *in vitro* by using pRNAT-U6 vector transfection with MSCs (185). In a study by Shang et al. in 2020 investigating the therapeutic effects of MSCs–exosomal miR-1231, two cell lines related to PC, namely, Panc-1, which had the highest expression of miR-1231, and BxPC-3, which had the lowest expression of miR-1231, were selected (185). After co-incubation with PCC-related cell lines, these exosomes fuse with them and transfer miR-1231 along with other exosome contents into them and change the actions of the cells. To check the efficiency of transferring this miR into cancer cells, the expression level of EGFR and cyclin E as its direct targets (186) was evaluated by immunoblots. The results showed that transferring this miR to PCCs leads to decreased proliferation ability (185). Moreover, the examination of wound-healing assays to investigate the migration ability of PCCs and the ability of invasion using Matrigel-coated Transwell chambers shows a significant decrease in migration and invasion in exosome-recipient cells (185). In addition, it has been demonstrated in this study that the adhesion of tumor cells to the matrix is also reduced in tumor cells receiving exosomes (185). In addition, the results of animal phase studies show that using exosomes with overexpressing miR-1231 in pancreatic tumor model mice leads to a decrease in the size, weight, and growth of tumors and an increase in survival in mice (185).

Another antitumor miRNA loaded in exosomes is miR-34a, whose efficacy has been evaluated in treating pancreatic cancer (187). The results of this study show that miR-34a loaded inside exosomes derived from HEK293 cells can reduce tumor growth in both *in vivo* and *in vitro* conditions (187). Bcl-2, an anti-apoptotic molecule that leads to increased tumor cell survival (188), is one of the direct targets of miR-34a (189). In PCCs receiving exosomes (Panc28 and Miapaca-2 cells), the amount of miR-34a is associated with a significant increase. Meanwhile, qRT-PCR results show that Bcl-2 expression in these cells is significantly reduced compared with the control group. At the same time, the expression level of pro-apoptotic proteins such as Bax and P53 has significantly increased (187). Moreover, Annexin-V/PI results show increased apoptosis in the group receiving exosomes containing miR-34a. In addition, the investigation of the effect of these exosomes *in vivo* confirms the results of the *in vitro* phase. It shows that the use of these exosomes in pancreatic cancer model mice leads to a reduction in tumor growth (187).

In another study, instead of drug loading in MSCs–exosomes, these cells were primed with paclitaxel (PTX) as an antitumor drug (190, 191). The results of this study show that this action can lead to the production of exosomes from these cells (murine SR4987 line) that carry this drug inside them and, after reaching the tumor cell and merging with it, deliver the drug to them. Using these exosomes on the pancreatic cancer-related cell line (CFPAC-1) decreased tumor cell proliferation (190).

## 5 Conclusion and future perspective

Exosomes are very important in investigating the conditions and characteristics of cells. Due to the higher production of exosomes by tumor cells, the amount of these vesicles in the blood of people with tumors usually increases, and the analysis of these exosomes can help in the early diagnosis of various cancers. It is worth noting that the content of the different types of tumor cell-derived exosomes is similar in many cases. This issue can be challenging in accurately diagnosing tumors by exosomes. On the other hand, exosomes can be used in the treatment of pancreatic cancer, and in general, various mechanisms that can lead to tumor cell survival, proliferation, signaling, and tumor-related immune inhibition can be targeted and inverted by exosomes. **Table 4** summarizes the studies in which exosomes have been used to diagnose and treat pancreatic cancer in ClinicalTrials.gov. Considering the problems related to the homogeneity in isolated exosomes, storage difficulty, and difficulty in large amount isolation of exosomes for treatment, this field of research requires further development and standardization of related methods. In addition, as shown in **Table 4**, most of the studies related to the therapeutic application of exosomes in pancreatic cancer are limited to laboratory and animal studies, and their road to the clinical phases requires studies related to safety.

**Table 4:** Exosome-related studies in the clinical trial phase.

Study title	Study type	Intervention model	Estimated enrollment	Intervention	Status	Phase	Last update	NTC number
iExosomes in Treating Participants With Metastatic Pancreas Cancer With KrasG12D Mutation	Treatment	Single group assignment	28 participants	Exosomes with KRAS G12D siRNA	Recruiting	Phase 1	2022	NCT03608631
ExoLuminate Study for Early Detection of Pancreatic Cancer	Diagnostic	Observational cohort	1,000 participants	N/A	Not yet recruiting	N/A	2022	NCT05625529
Diagnostic Accuracy of Circulating Tumor Cells (CTCs) and Onco-exosome Quantification in the Diagnosis of Pancreatic Cancer -PANC-CTC	Diagnostic	Observational cohort	52 participants	N/A	Completed	N/A	2018	NCT03032913
Circulating Extracellular Exosomal Small RNA as Potential Biomarker for Human Pancreatic Cancer	Diagnostic	Single group assignment	102 participants	Venous sampling	Unknown	N/A	2020	NCT04636788
New Biomarkers in Pancreatic Cancer Using EXPEL Concept (PANEXPEL)	Diagnostic	Observational cohort	200 participants	N/A	Recruiting	N/A	2022	NCT03791073

## Author contributions

KJ was responsible for the conception and design of the study and for the invitation of co-authors to participate in the study. XF and HL wrote the original manuscript draft. KJ and JQ reviewed and edited the manuscript critically for important intellectual content and provided comments and feedback for the scientific contents of the manuscript. All authors read, revised, and approved the final manuscript.
